# Role of "external facilitation" in implementation of research findings: a qualitative evaluation of facilitation experiences in the Veterans Health Administration

**DOI:** 10.1186/1748-5908-1-23

**Published:** 2006-10-18

**Authors:** Cheryl B Stetler, Marcia W Legro, Joanne Rycroft-Malone, Candice Bowman, Geoffrey Curran, Marylou Guihan, Hildi Hagedorn, Sandra Pineros, Carolyn M Wallace

**Affiliations:** 1 Independent Consultant, 321 Middle St., Amherst, MA 01002, USA; 2 VA Puget Sound Health Care System, Health Services Research & Development, Met Park West, 1100 Olive Way, Suite 1400, Seattle, WA 98101 USA; 3 Reader in Health Services Research, Centre for Health-Related Research, University of Wales, Bangor, UK; 4 VA San Diego Healthcare System, QUERI-HIV, Health Services Research & Development, 3350 La Jolla Village Drive (111N-1), San Diego, CA 92161 USA; 5 Central Arkansas Veterans Healthcare System, Center for Mental Healthcare & Outcomes Research, 2200 Fort Roots Drive, Building 58 (152/NLR), North Little Rock, AR 72114 USA; 6 Midwest Center for Health Services and Policy Research, Edward Hines, Jr. VA Hospital (151-H) Hines, IL 60141 USA; 7 Minneapolis VA Medical Center, One Veterans Drive, Minneapolis, MN 55417 USA

## Abstract

**Background:**

Facilitation has been identified in the literature as a potentially key component of successful implementation. It has not, however, either been well-defined or well-studied. Significant questions remain about the operational definition of facilitation and about the relationship of facilitation to other interventions, especially to other change agent roles when used in multi-faceted implementation projects.

Researchers who are part of the Quality Enhancement Research Initiative (QUERI) are actively exploring various approaches and processes, including facilitation, to enable implementation of best practices in the Veterans Health Administration health care system – the largest integrated healthcare system in the United States. This paper describes a systematic, retrospective evaluation of implementation-related facilitation experiences within QUERI, a quality improvement program developed by the US Department of Veterans Affairs.

**Methods:**

A post-hoc evaluation was conducted through a series of semi-structured interviews to examine the concept of facilitation across several multi-site QUERI implementation studies. The interview process is based on a technique developed in the field of education, which systematically enhances learning through experience by stimulating recall and reflection regarding past complex activities. An iterative content analysis approach relative to a set of conceptually-based interview questions was used for data analysis.

**Findings:**

Findings suggest that facilitation, within an implementation study initiated by a central change agency, is a deliberate and valued process of *interactive problem solving *and *support *that occurs in the context of a recognized need for improvement and a supportive interpersonal relationship. Facilitation was described primarily as a *distinct role *with a number of potentially crucial behaviors and activities. Data further suggest that external facilitators were likely to use or integrate other implementation interventions, while performing this problem-solving and supportive role.

**Preliminary Conclusions:**

This evaluation provides evidence to suggest that facilitation could be considered a distinct implementation intervention, just as audit and feedback, educational outreach, or similar methods are considered to be discrete interventions. As such, facilitation should be well-defined and explicitly evaluated for its perceived usefulness within multi-intervention implementation projects. Additionally, researchers should better define the specific contribution of facilitation to the success of implementation in different types of projects, different types of sites, and with evidence and innovations of varying levels of strength and complexity.

## Background

Implementation of research findings into practice is a complex undertaking that has often fallen short of expectations. In part, this is due to the current lack of substantive knowledge regarding both individual implementation interventions and the interrelationship of multiple interventions used in many studies [1-4].

One type of intervention often used in conjunction with other implementation interventions is that of a change agent. A *change agent *is an "an individual who influences clients' innovation decisions in a direction deemed desirable by a change agency [p.28, [5]]," with *change agency *defined as an "organisation or other unit that promotes and supports adoption and implementation of innovations [p. 372, [6]]." Specific types of change agent roles typically studied in recent health care implementation research include opinion leaders, outreach educators, academic detail workers, and clinical champions.

An additional change agent role posited as key to successful implementation is that of a *facilitator *[7,8]. The PARIHS framework, developed from relevant literature and case studies, posits that successful implementation is a function of three factors: the nature of the evidence, quality of the context, and facilitation. Facilitation was recently highlighted within the United States (US) Veterans Health Administration (VHA) as theoretically-promising to the change agentry role of QUERI. [See Table 1 for a definition of QUERI and other key terms.] At the conclusion of an initial round of projects, several QUERI implementation researchers decided to collaboratively, retrospectively, and systematically explore their facilitation-related experiences to better define the concept and its potential value.

**Table 1:** QUERI description & key definitions

*QUERI *is an improvement initiative wherein participating researchers are expected to simultaneously study the implementation process and work toward rapid and significant improvements in the health of veterans, in terms of specific diseases and related problem areas. It was initiated in 1998 and is described as a comprehensive, data driven, outcomes-based and output-oriented quality improvement effort that focuses on the rigorous application of best clinical practices into routine care/systems [15] The term QUERI is used to refer to this overall VHA initiative and its national leadership, as well as to specific QUERI teams that are organized around a disease or other issue-related entity (e.g., the Mental Health QUERI and the Spinal Cord Injury QUERI).
*External facilitation *refers to facilitation that comes from a change agency outside of the implementation site; in this case, from a QUERI study team.

*Implementation intervention or implementation tool *is defined as a single method or technique to facilitate change and, thereby, adoption of best practice recommendations, e.g., an opinion leader, electronic clinical reminder, or interactive education program. These also are referred to as "uptake," "adoption," or "change" interventions.

*Implementation strategy or program *is defined as an integrated set (bundle, package) of implementation interventions. QUERI implementation studies typically evaluate implementation strategies or programs rather than individual interventions, in that such an intervention is frequently insufficient to achieve implementation in complex clinical settings.

*Internal facilitation *refers to facilitation that would, if present, come from within an implementation study site.

### The PARIHS framework, facilitation and relevant research

The PARIHS framework, which was the basis for initial use of facilitation in QUERI, conceives of facilitation as one of the three factors key to successful implementation [7,8]. The originators of the framework suggest that *facilitators *have a key role in helping individuals and teams understand what they need to change and how to change it to successfully implement evidence into practice [8]. As such, facilitation can be identified as a potential intervention that enables the implementation of evidence into practice [9]. However, the precise role of facilitation and its contribution to the success of implementation, particularly within multi-site projects across a health system, has yet to be thoroughly described, operationally defined, or well-evaluated.

The process of facilitation and the related role of a facilitator are evident in a number of different fields and disciplines ranging across education, counseling, management, clinical supervision, and quality improvement. Available evidence reviewed by Harvey and colleagues [9], however, illustrates variable interpretations and approaches to facilitation. For example, in practice-based learning methods, such as clinical supervision, the focus is on "facilitating experiential learning through critical reflection, dealing with psychological defensiveness and challenging cultural norms [p.580, [9]]." In contrast, in fields such as quality improvement the purpose of facilitation appears to be more oriented to task and goal achievement. The purpose of facilitation can thus vary, ranging from "providing help and support to achieve a specific goal" to "enabling individuals and teams to analyze, reflect and change their own attitudes, behaviours and ways of working [p.580, [9]]." Facilitation also may occur in variable forms, e.g., as a facilitator internal to an implementation site, an external facilitator [i.e., QUERI change agent], or a combination thereof. In the latter instance, an external facilitator might work with an internal facilitator to develop his/her facilitation skills and abilities and/or help to develop an enabling context. Finally, facilitation has been described as an appointed role that encompasses a wide range of techniques and approaches [9].

Facilitation, as described to date, is a complex and multi-faceted concept. Based on the current state of the evidence, questions exist as to both: 1) Whether facilitation is conceptually and operationally discrete from other change agent interventions, such as educational outreach, and 2) How facilitation relates to other types of interventions or processes within a multi-faceted implementation project [9]. Aspects of educational outreach, for example, appear in some facilitation models but not others, and, as Rycroft-Malone, et al[10] noted recently, there is a potential overlap of project management and facilitation. Additionally, there is a lack of common intervention definitions within the implementation field, as well as a failure at times to differentiate planned versus actual operationalization of those interventions [11,12]. This makes comparisons across studies difficult and adds to the lack of clarity of any implementation concept, including facilitation. Further questions arise from the following:

• Use of internal, external, and/or a combined approach to facilitation – again, at times without specification or recognition of actual activities and divided responsibilities; and

• Use of an external facilitator, evident in QUERI, working across multiple and variable implementation sites (units and/or facilities) from a central location within a health care system.

In summary, findings from evaluative studies reflect the diverse ways in which facilitation has been conceptualized and applied. This makes it difficult to combine the findings to draw meaningful conclusions about the nature and effectiveness of facilitation as a distinct implementation intervention. According to Harvey and colleagues, the findings from qualitative and quantitative studies do suggest that a facilitator with face-to-face communication and using a "range of enabling techniques has some impact on changing clinical and organisational practice, although the effect size is variable and associated with differing costs [p.585, 9]." Exactly what enabling techniques influence change, with which type of facilitator, using what type of approach, or in what type of setting or context remains unclear.

### Purpose of the evaluation of QUERI facilitation experiences

The completion of a first wave of implementation projects within QUERI provided an opportunity to explore the concept of facilitation and highlight essential details of the facilitator role. (See additional file 1, an Adobe Acrobat 7.0 PDF document: *Study Projects per QUERI Team: Related Goals and Implementation Interventions *for a description of the multi-intervention strategy for each QUERI study. Although facilitation was present at varying levels in all the projects, it had not been recognized at the initiation of these projects as a distinct activity or intervention. This type of approach further highlights problems associated with making sense of multi-faceted interventions and their related effects.)

The overall purpose of this evaluation was to raise consciousness about the need to better understand the concept of facilitation – and thereby enhance its deliberative operationalization and encourage its explicit evaluation in future QUERI research. More specific evaluative objectives (see Table 2 for details) covered two aims: 1) To clarify the precise nature of facilitation as a potentially discrete versus diffuse intervention within an implementation strategy, and 2) To uncover critical issues requiring future research.

**Table 2:** Specific objectives of the QUERI evaluative project on facilitation

1. Provide insights based on accumulated experiences regarding the following:
a. The operational nature of external and internal facilitation, across multiple sites and across multiple implementation research projects, within the VA.
b. The differentiation of facilitation from other change agent-related implementation activities, such as opinion leadership, as well as other relevant implementation interventions.
c. Facilitators of and barriers to facilitation.
d. The potential role of facilitation (both external and internal) as a distinctly separate implementation intervention.
e. The essence of facilitation as a role or function.
i. Within a QUERI implementation study: Is it one function among many played by an Implementation Research Coordinator*? Do others on the study team also play that function?
ii. Within a VISN:** Is it a role or function that might conceivably support the routine uptake of evidence?
2. Identify critical research questions regarding facilitation relative to the above insights.

*Implementation Research Coordinator: The QUERI team member whose primary responsibility it is to ensure inclusion of educational, social, behavioral, and organizational sciences when the team is planning and executing implementation research. This person also often plays the role of the external facilitator.

**VISN: Veterans Integrated Service Networks; the regionalized, clinical structural organization of the Veterans Administration's health care delivery system; there are 23 such Networks.

The remainder of this paper contains a description of the reflective method used to identify and analyze QUERI facilitation experiences, a set of summarized findings with detailed tables, and associated research implications.

## Method

### Approach

A reflective exploration was the method chosen to gain in-depth insights into the nature of facilitation across multiple VA research projects. A reflective exploration is a process that systematically stimulates and supports recall and analysis of a past complex experience; in this case, involving interviews of a purposefully selected group of implementation research change agents. The approach was based on Kolb's experiential 4-phase learning cycle [13]. As applied to this study, learning cycle phases paralleled data collection and analysis processes, and progressed as follows:

• Review of the concrete experience of facilitation, through preparatory work and a structured interview;

• Conceptual reflection on that experience, in terms of specific conceptual issues identified in a question-based analysis; and

• Participation in a reflection on synthesized conceptualizations and generalizations regarding facilitation, developed through thematic analysis and cross-interview synthesis.

[See additional file 2, an Adobe Acrobat 7.0 PDF document: *Reflective Questions and Interviewee Preparatory Work*.]

A possible fourth phase was not part of this study (i.e., testing implications of concepts in new situations). However, selected questions were framed in a future perspective. For example, what should have happened in the implementation study? What might have been more effective? And by implication, what should be done in the next implementation study?

### Data collection method

An initial, one-to-two hour semi-structured telephone interview was conducted with each participant. An implementation expert carried out the interviews (CBS), which primarily captured qualitative data, while another team member (ML) served as the note taker (except for her own interview, the pilot) and participated in the interview if clarification was required. Guided by the Kolb framework [13], the reflective interview process focused on exploration of the following topical areas:

• The nature of facilitation; its purpose, role, and function

• Facilitators and barriers to facilitation

• Factors distinguishing facilitation from other change agent roles and implementation interventions

• Other influencing factors.

The initial interview was followed by a series of shorter interviews and e-mail communications over approximately 7 months to obtain the iterative participant reflections required by the learning cycle. [See additional file 2: *Reflective Questions and Interviewee Preparatory Work*, for the detailed interview guide.]

### Participants and sites

Seven VA researchers from six QUERI-related VA implementation projects agreed to participate in the evaluation. All participants had been involved in explicit or implicit facilitative processes, occasionally as a supporter and supervisor of a facilitator.

Each QUERI project in which these interviewees had been involved was designed with a goal of improving a specific aspect of health care for a specific group of veterans. [See additional file 1: *Study Projects*.] The number of clinics or study facilities involved in an individual QUERI project ranged widely – from 4 to 23. Each interviewee participated in the execution of one of these projects as a study team member and facilitator (or facilitator supervisor/supporter).

The evaluation plan was submitted to the Human Subjects Division at an organizer's home site (ML) and was considered to be a QUERI quality review. The manuscript was more recently presented to the same Human Subjects Division (HSD) relative to its proposed publication in the health care literature. In consultation with the University of Washington Institutional Review Board Chair, the HSD concluded that this work did not constitute human subjects research and, therefore, did not need a certificate of exemption. Nonetheless, specific ethical principles regarding confidentiality and protection of data were adopted. For example, it was agreed that the organizers of the evaluation (CBS, ML, JR-M) would be the collectors and analyzers of raw data and would maintain its confidentiality; individual experiential data would be synthesized across participants, so individual participant experiences and perceptions would be anonymous; and quotations used in any outputs would not include information that identified the participant.

### Analysis process

Following each interview, a detailed process was followed to enhance reliability and trustworthiness of data acquisition, analysis and interpretation. Table 3 describes the iterative process used to affirm accuracy of recorded experiences, individual case interpretations, and cross-interview syntheses. Interviewee participants received a draft transcript of their own interview to ascertain whether they believed it to be a true record and were invited to contribute any additional thoughts since the interview. Next, interviewers organized and synopsized the raw experiential report within each finalized interview to reflect an interviewee's core, conceptual response to each question. This draft analysis was provided to the participant for further comment and affirmation or challenge.

**Table 3:** Reflective analysis process for experiential data

1. The transcribed text from the structured, interview-based reflection was affirmed or modified to create an accurate description of an interviewee's facilitation experience through the following steps:
• Initial agreement between the interviewer (CBS) and recorder (ML),
• Review of this "cleaned" recording by the interviewee, and
• Revision by the interviewer/recorder until agreement was reached with the interviewee.
2. One of the implementation experts performed an initial question-based conceptual analysis of each individual interview (CBS), after which the interviewer/recorder reached consensus as needed, followed by two additional steps:
• Conceptual review of this product by the interviewee for its affirmation or alternative interpretations as needed, and
• Revision by the interviewer/recorder until agreement was reached with the interviewee.

3. The interviewer, recorder and a member of the PARIHS framework team (JR-M) reached consensus on a final thematic analysis and cross-case synthesis relative to pre-established exploration questions across the accumulated interview data.
• Review of this product by each interviewee,
• A final telephone discussion with each interviewee to obtain general consensus on the content/format of the report, and
• Review of final draft by all members of the team, with final decisions based on common perceptions.

As data were primarily qualitative, content analysis, whereby data are broken down and subsequently built up into themes, was employed following Huberman and Miles [14]. In this case, responses across interviews were clustered in relation to the pre-determined questions to identify similar or distinct perceptions and experiences. Common responses or themes across cases were identified. Interviewee quotes were linked to draft themes for illustrative and clarifying purposes, and, where relevant, distinct experiences that differed from the majority were highlighted. Throughout the analysis process, the third organizer [JR-M] acted as a critical reader, challenging emerging themes and hypotheses, and ensuring re-formulation of ideas where appropriate.

Based on consensus of the interviewers [CBS & ML] and evidence-based clarifications requested by JR-M, the draft of this cross-case analysis was circulated to all participants. Affirmation or suggestions about alternate interpretations or wording was requested during individualized phone calls. Overall, participants reviewed and affirmed the product at each step of the analysis, including the summary of the cross-case synthesis.

## Findings

Study findings are presented in terms of key issues that emerged relative to the evaluative questions. In reading the findings, the following context is of note:

• Implementation was being driven by a central agency within the VA health care system external to practice sites and was focused on creation of standard evidence-based practice (EBP) throughout the system.

• The PARIHS model was familiar to many of the investigators but was not used prescriptively; it also was not the only potential definition of facilitation available to the participants or QUERI teams.

• The concept of facilitation had to be operationalized by each QUERI project, as none were given a detailed, operational set of guidelines for the emerging role.

• Most projects were placed under a tight timeline for demonstrating rapid, targeted improvements in current practice.

• Findings relate to an "external" facilitator role as opposed to an "internal" facilitator role. [See Table 1.] Local individuals directly involved in or assigned to an active local role in a QUERI implementation project, and interacting with the external facilitator, are henceforth referred to as "internal change agents." Internal change agents within implementation sites were variously termed by QUERI project teams as a clinical champion, opinion leader, site coordinator, site leader, or site team leader – but not as a facilitator. As one interviewee noted, these internal players were expected to implement the new practice and "*figure out how to ensure that patients received the recommended care*." In some projects, the person filling this internal site role was a unit manager or held another formal leadership role; in many projects it was a physician; and in others, the external facilitator worked with "*emergent groups*" or "*different individuals for different interventions," *as each required a particular skill or role.

### The nature of facilitation

As experienced by these VHA implementation researchers, facilitation is a valuable and critical process of interactive problem-solving and support, which occurs in the context of a recognized need for improvement and a supportive interpersonal relationship. The recognized need is one derived through research of best practice and diagnostic analysis of a site's performance gaps [15].

In general, interviewees viewed the end goal of facilitation as helping people in health care settings modify their work to incorporate a specific evidence-based clinical practice. This "helping" effort was seen to occur in general through the support of locals' use of an evidence-based implementation strategy – originally developed by the research team (additional file 1: *Study Projects*) – and through provision of other assistive and encouraging activities.

More specifically, interviewees identified the objectives of external facilitation as follows:

1. To help internal change agent/s at implementation sites understand what needs to change and how change can occur. This involved identifying organizational or provider factors that could make it easier or harder to achieve the adoption of a best practice; providing information regarding the evidence, related changes and/or useful problem solving linkages; and assisting in the reduction of identified barriers to progress.

2. To provide support to internal change agent/s in the form of encouragement, mentoring, and positive feedback, as appropriate.

The following individual participant comments highlight these objectives ['*you' *and '*them' *herein refer to the internal change agents as applicable]:

• *"We can help you with problems and can help explain the implementation interventions to you; these are the tools that will help you, and we can help you use them."*

• *"Facilitation leads to enabling staff at the sites put the interventions in place, and to maintain and/or modify them over time."*

• *"The essence of facilitation is trying to make things easier or easiest to make changes. To help them understand how they need to change, give them tools, monitor, and keep providing support as necessary."*

### Facilitation and other roles

A key question about facilitation is whether it is different from or part of other implementation interventions, processes or roles. Based on the experiences of these QUERI interviewees, the following observations were made about facilitation:

1. In its most concrete form, i.e., as an implementation change agent or facilitator role, facilitation can be viewed as a distinct intervention. However, in general interviewees suggested that facilitators will likely use or integrate other implementation interventions while performing this problem-solving/supportive function. For example, they might provide education to enhance an internal change agent's knowledge about the targeted evidence and its credibility. The difference between education and facilitation is, one suggested, related to "*whether you are using predetermined materials, identified at the beginning of the project or whether you discover a need and develop or modify preexisting information for the use of those with the problem. You might even develop the education to meet requests of the local personnel." *Another said, "*On the site visit, I came in with a PowerPoint presentation. That is education. When they called me for help ... that was different. It was facilitation."*

Facilitators also might help internal change agent/s understand and utilize audit and feedback data provided by an implementation research team, or use persuasive advocacy/championing to reinforce the need for a targeted evidence-based change in relation to local practice gaps. In terms of the latter, another participant suggested, "*Facilitation is not equivalent to marketing ... or networking. But, facilitation can encompass those particular roles and be part of making these roles easier." *Yet another said, *"It really has a lot to do with creating buy-in/activating the sites/championing the recommendations."*

Two interviewees described their view of the distinctness of facilitation as follows:

• *"Facilitation is more general than other change roles – more flexible. I think facilitation is a concept that addresses change and the individuals who will create the change. Its precise character and activities will depend on the purpose of facilitation, the structure in which people work, and the people with whom the facilitator will work."*

• *"Facilitation is more two-way than other implementation strategies, not as prescriptive, and is more adaptive and respectful of what is in place."*

2. Facilitation can occur at multiple levels within an implementation project. Multiple individuals can contribute to such facilitative support in formal or informal ways. Clinical leaders, for example, might help to remove a barrier to change or a study team member (i.e., other than the facilitator) can assist with a needed linkage to an outside expert or a problem solver.

3. Facilitation may be considered a *mediating *implementation intervention or process [4] because it often enables and supports actualization of other implementation interventions, such as electronic clinical reminders, audit and feedback, or operational system changes. In reviewing the final manuscript, one participant described it as the "*engine that drives the other *[implementation] *interventions *... [an] *ingredient that makes them work*."

4. Within the context of action-oriented implementation research such as QUERI [15], formal facilitation was seen to begin when the external facilitator starts to establish a working relationship with an internal change agent. Prior to that point, as a member of the research team, facilitators may participate in pre-implementation tool development or other groundwork. As one participant explained, "*I did a lot of the developing of the implementation interventions and related educational materials... this was not a part of my facilitation role." *This facilitator also described how "*my work changed from education to facilitation over time*." Another interviewee talked about the difference between facilitation and "*getting them ready*" for implementation, and included educational outreach activities as part of the latter groundwork.

5. The study team project management role within QUERI was often separate from the facilitator role, or at the least was seen as a separate set of functions. In addition to pre-implementation groundwork such as decision-making about interventions, diagnostic analysis of targeted sites, development of toolkits, or engagement of leadership, QUERI project management often focused on data collection, report development, scheduling of team meetings and record maintenance.

### Key components of external facilitation

As noted above, facilitators appear to help internal change agents actualize an implementation plan and, thereby, EBP through two key components of the facilitation process, i.e., *interactive problem solving *and *support*. A summary regarding distinct behaviors and activities related to these two components of the external facilitator role are described below, with further detail provided in Tables 4, 5, and 6.

**Table 4:** The external facilitator role in problem identification/resolution: potential activities/behaviors per QUERI experiences

*1) Problem identification and resolution*
a) Identification and clarification of problems related to implementing both the evidence-based practices and related implementation interventions
i) Provides and reviews with the ICA (internal change agent) information on current gaps, identified barriers, and other feedback
ii) Helps ICAs understand their own situation and the nature of problems within their culture/context/work language.
(1) Note: Requires the external facilitator to assess and understand the local context.
iii) Defines and frames a user's problem in a way that the ICA can best deal with it.
b) Review of potential approaches for problem resolution
i) Shares viable solutions/options with ICA/s.
(1) Seeks information and answers within the greater VA system, including other implementation sites.
(2) Works with the QUERI project team to help develop viable alternative activities to solve site problems and remove complex barriers.
ii) Helps ICA/s figure out appropriate strategies to address barriers.
iii) Creates opportunities for resolution/actions by the ICA, e.g., by:
(1) Identifying experts,
(2) Identifying peer sites,
(3) Identifying resources in the VA, and
(4) Establishing a link between the ICA and potential problem solver/s in VA.
iv) Negotiates appropriate solutions with the internal agent, as needed.
c) Assistance in setting clear goals.
i) Helping ICA/s set realistic goals to overcome problems and achieve evidence-based practice targets.
d) Direct implementation or initiation of solutions in relation to both identified local site needs and the need to see core QUERI intervention strategies implemented:
(1) When specific expertise or skill is required,
(a) Provides more education, e.g., re: the implementation strategies or the targeted evidence, and
(b) Generates needed tools or sample materials.
(2) When specific networking or external contacts are required,
(a) For example, obtains available resources for the ICA/s or sites.

**Table 5:** The external facilitator role in communication and formative use of data: potential activities/behaviors per QUERI experiences

*1) Communication activities/behaviors*
a) Provides a basis for regular, goal-focused contact.
i) Establishes multiple means of one-way and two-way communication with ICA/s [internal change agent/s]: e.g., e-mail; phone conferences, discussion groups, phone contact information, and a problem-focused newsletter.
ii) Obtains information to keep the QUERI team updated.
b) Provides clarity and an information source for the ICAs:
i) Shares knowledge regarding QUERI implementation interventions,
ii) Shares knowledge regarding the VA system, and
iii) Shares knowledge regarding change processes.
c) Structures and leads regular communication across study sites regarding, e.g.:
i) Status of implementation efforts,
ii) Successful problem solving approaches for various issues, and
iii) Similar roles and problems.
d) Establishes linkages for ICA problem-related actions:
i) Helps them frame questions to ask of key resources.
e) Intercedes with VA leadership (internal or external) on behalf of ICA.
*2) Formative use of data activities/behaviors*
i) Reviews diagnostic information in order to understand the local context.
ii) Monitors/tracks and uses progress data:
(1) For example, regarding goals and both intermediate and end result outcomes.
iii) Monitors/tracks and uses problem data:
(1) For example, regarding issues/barriers.
iv) Monitors ICA activities to know what is happening:
(1) Monitors use of new solutions for site problems, and
(2) Identifies needs and issues of an ICA.
v) Monitors and uses data re: the value of and need for external facilitation.

**Table 6:** The external facilitator role in a supportive relationship: potential activities/behaviors per QUERI experiences

*Establishing and maintaining a supportive relationship:*
a) Maintains multiple means of contact and accessibility with the ICA.
b) Provides rapid responses to ICA requests, as feasible.
c) Provides reassurance and encouragement:
i) Provides information on progress,
ii) Provides cheerleading,
iii) Provides psychological support, and
iv) Enables peer-based social support.
d) Empowers ICAs – sets the stage for them, gives them permission to do things on their own.
e) Serves as a "nudge" and a source of external expectation for progress.
f) Makes required actions quick and easy, when possible.
g) Mentors and develops skills in the ICA, as needed:
i) Shares knowledge,
ii) Teaches skills,
iii) Enables ICAs to solve their own problems, where feasible, and
iv) Provides role feedback.

#### Problem solving

The QUERI facilitator was described by participant accounts as engaging in interactive, contingent problem solving. QUERI facilitators were commonly depicted as a problem solving resource for internal change agents, with the intent to enable these internal agents to solve their own problems. *Facilitator *– *internal change agent *problem solving was seen as *interactive *because of the need for ongoing dialogue and collaboration, and as *contingent *in terms of the pre-determined evidence-based goal of the QUERI project, the complexity of involved changes, and the potentially differing needs of different sites.

Analysis of participant accounts resulted in identification of three enabling and *inter-connected *aspects of problem-solving, as illustrated below:

1. *Problem identification and resolution *(Table 4): This usually involved helping the internal change agent/s in each site identify local problems and viable solutions, which often varied from site to site. As three of the participants described it: "*Sometimes people were having trouble and we helped them name the problem so we, or they, could seek help"; "It involves helping others to get whatever they are trying to do done ... to help with the process... helping them through barriers, talking them out, and giving advice"; *and *"We facilitators engaged these players to identify problems and then go solve it themselves."*

Occasionally the external facilitator would take direct action (i.e., the "doer/task function" in the PARIHS model [7,8]) but only in particular circumstances. For example, the facilitator might take such actions in the case of a problem requiring special expertise, or for role-modeling purposes. As one participant explained, "*Facilitation focused on the idea of doing for and then they would do."*

2. *Use of formative data *(Table 5): An inherent component of problem identification and, at times, resolution is the acquisition, review and use of data. QUERI external facilitators obtained and used multiple types of formative data, which they may or may not have collected themselves. They used such information to enhance their ability to make needed changes at the site, provide real-time feedback to internal change agent/s, and understand and fulfill their own role. Examples of such formative data are as follows:

• Status of pre-requisite implementation factors in the local context, i.e., clinician perceptions regarding credibility of available clinical practice guidelines;

• How implementation was progressing from a fidelity/integrity of innovation [11] and activity point of view, i.e., the degree to which the internal change agent/s and others actually did institute identified components of the implementation strategy;

• How implementation was progressing in relation to outcomes, e.g., the degree to which clinicians had adopted recommended guidelines; and

• The nature of local factors that appeared to be essential to the spread of implementation and progress, e.g., the degree of visible leadership support or cooperation of needed departments.

As individual participants said, "*The facilitator worked to gather data from them about barriers and problems"; "I asked questions about the local context and sought to understand it"; *and [At the start of implementation] *"I made site visits to learn about current clinic practices including, for example, what they were doing about *[*X*] ...*by whom, how often, when.... And *[I] *did give them a lot of feedback on their practice once they started the intervention."*

3. *Communication *(Table 5): As the link between the study team, implementation sites, and other relevant stakeholders, external facilitators supported the exchange of information through multiple communication channels. Thus, facilitators tried to establish a means of regular, goal-focused contact and became a resource and boundary spanner [3,6] for ongoing information exchange or networking. As individual participants said, *"If the opinion leaders needed input from *[another research team member], *they went through the facilitator"; "I could tell them how other sites did it – give them information about solving problems"; *and *"Sometimes I could put them in contact with other clinics that were doing well *... [and] *I collected protocol books from all the clinics, put them together, and shared it across clinics so they could decide what to do." *Such communication activities appeared to indirectly assist sites to solve problems and to provide mechanisms for moral support among internal change agent peers.

#### Support

One interviewee described the essence of facilitation as "*support and encouragement *– *more of a relationship where you work with the team or identified person at the site, rather than an outsider coming in with educational materials." *Other interviewees described encouraging and helping internal change agents [ICAs] feel that they – the ICAs – could actualize implementation and, thus, enhance adoption of the targeted evidence. For example, they talked about *"allowing and encouraging them to do their best to achieve these goals"; *and *"You have to share that sense of goal achievement ... attainment in order to get there, in helping them. It means cheerleading."*

Overall, external facilitators tried to focus on enhancing the ability of ICAs to succeed, strengthening their sense of accomplishment, and reinforcing the fact that assistance was at hand. Table 6 lists in more detail the essence of establishing and maintaining a reciprocal, supportive, problem-oriented interpersonal relationship, as experienced by these interviewees. This support function also appeared to involve helping ICAs understand what is required to facilitate, which may involve clarifying expectations for both the external change agency facilitator and the ICA, and keeping these expectations realistic.

### Factors related to the perceived degree of success of external facilitation

There are barriers to and enablers of external facilitation, at times reflecting either the absence or presence of the same critical factor. Table 7 depicts this relationship in terms of common themes across interviewee experiences and sample quotes illustrating contrasting circumstances.

**Table 7:** Barriers and facilitators of external facilitation per QUERI experiences

Facilitators*	Barriers
*Internal To The Implementation Sites*	

• Motivation for implementation	• Lack of motivation for implementation
• Supportive leadership	• Lack of leadership support

*External to the Implementation Sites*	

• Sufficient contact with ICA**	• Insufficient contact with ICA
• QUERI team that understands and supports the role	• Lack of understanding or operationalization of the role by the QUERI team
• Facilitator skill/experience/attributes	• Inadequate facilitator skill/experience/attributes

*Sample Interviewee accounts*	

• "*Some *[sites] *wanted to change and were ready ... this was a high priority for them*."	
• [A QUERI leader]... "*being protective so the facilitators could do facilitation rather than project work...more explicitly, s/he 'made space' for the facilitators*."	◦ "*The concept of facilitation really wasn't thought about in a formal manner *... *Data was more on the mind of the study team*."
•" *My training and background in group dynamics... and ability to be flexible*."	
• "*I know it would be cost prohibitive to have an external person at every site, but that would be best. The teams need an external person to prod and answer questions*."	◦ "*Not being able to be there; not knowing what they are going through is a problem*"
• "*You need to ID the skills you need and hire the people that fit that profile and that people like. The facilitator needs to be really familiar with the work to be done, have charisma, and be able to inspire people to change*."	◦ "*Lack of motivation; lack of activation; lack of buy-in by lead provider*."
• "*The most important thing is the level of buy-in of the site lead provider. True of all cases. They always set the tone*."	◦ "*Only working with very busy docs – it was an up hill battle. ...We felt 'in the dark' about what was happening. We hope that having nurses ... and clerks in this role, there will be more eyes and ears*."

*The factor was present OR perceived that it would have been helpful if it had been present.

**ICA = internal change agent.

Four key factors and related observations were described as follows:

1. *Motivation/leadership at the sites: *These interrelated elements came in the following forms:

• An identified or assigned individual at the site, i.e., the internal change agent, who needed both commitment and time to put into the change process.

• Buy-in, or conversely, lack of support from formal administrative and clinical leaders for the change initiative and internal change agent role/s. Active leadership buy-in and support was observed, e.g., through provision of needed resources, verbal reinforcement of the importance of the initiative, and integration of changes into routine QI structures. Conversely, lack of support was noted in, e.g., a lack of responsiveness to needed assistance from key departments.

2. *Research team understanding and support of the external facilitator role*:

• Facilitation was a new concept to many of the projects and not uniformly understood. However, some teams were reported as having supportive members, such as those who recognized facilitation as a distinctive role that was critical to the team's work. An example of this support was the perceived protection of the facilitator's time to "facilitate."

• Study teams that did not make facilitation a distinct or inherent part of the project were perceived as having made support and communication with sites more difficult, thus impeding optimization of implementation. As one participant further explained, *"The problem was with how the implementation project was organized and the emphasis on the outcome rates. If anything, I think team members thought that telling staff to 'do something' would suffice to 'implement' the interventions."*

• It was agreed that both the implementation team and external facilitator need the latter's role to be explicitly defined, with a core of facilitation responsibilities and behaviors.

3. *Physical aspects of the facilitator role*: Overlapping with factors cited above is the element of *maintaining contact *between the external QUERI facilitator and internal change agent/s.

• In order to fulfill the interactive problem solving and support function, interviewees felt they needed regular communication, face-to-face contacts, and, at times, onsite presence to attend meetings or directly observe local discussions.

• In contrast, various interviewees reported not only inaccessibility problems with internal change agents, but also physical barriers of geographical distance, a large number of sites and thus a large number of internal change agent/s, and the prohibitive cost of travel. For example, external facilitators reported difficulty teaching interactive skills at a distance or evaluating related achievements.

4. *Selection and assignment of an individual to the external facilitator role*: Interviewees reported that individuals could have an easier or harder facilitator role depending upon their skills, experiences, and/or personal attributes. In general, they felt that an external facilitator had to be able to develop positive relationships with individuals and teams, have skills in problem solving, and have credible, relevant experience. Such experience might relate to time within the VHA system, a previous facilitator role or perhaps, in complex clinical situations, an applicable clinical background.

A multi-faceted interview question was used to assess participant views regarding the degree of "success" of facilitation efforts. Data are from six interviewees, as one felt s/he couldn't judge success. Interviewee perceptions regarding the success of individually identified and critical facilitation *behaviors *ranged on a scale from 1 (*not at all effective*) to 5 (*very effective/successful*). In terms of the success of the *overall *facilitative effort, on the same scale, two individuals rated facilitation as not effective ("1" and "2" respectively), while remaining summary ratings averaged 3.25. Perceptions of the overall success of individual QUERI implementation projects were similar, with an average rating of 3.4.

The above responses usually were qualified, in that success was said to vary across sites within a project. For example, one participant with an overall negative rating said that it varied from "*...site to site because of the intervention and the context. It differed by how much trust the team members at a facility gave me. They didn't all see me as a problem solver."*

Only one interviewee reported a significant degree of formal facilitation measurement. That analysis suggested "*More facilitation yielded better results – except for one site," *which may have had frequent contact due to resistance.

In those facilitation relationships perceived by interviewees to be more successful, one or more of the following factors, consistent with the above barrier/enhancer discussion, were likely to be cited:

1. External facilitators were perceived by internal change agents as having certain attributes, i.e., they were:

• Credible to the internal change agent/s, e.g., seen as understanding the evidence;

• Good communicators, i.e., open to being contacted, friendly, and outgoing, and having established good rapport; and

• Flexible and responsive to the needs of the internal change agent/s, i.e., either having answers to questions or being able to find them.

2. The external facilitator had a history of relevant experience, needed skills, and a commitment to facilitation.

3. The external facilitator was based in a QUERI team that from its inception recognized the value of facilitation and supported the facilitator's ability to be successful.

Some illustrative comments from three interviewees included the following: "*They said I was easy to communicate with, readily available. I returned calls. I either had answers or would find them." *Another was said to *"...have had a great rapport," *and a third felt that a facilitator had to be "*...someone who is well-liked." *In terms of a clinical background, one said, "*With no clinical background, it is difficult to talk to MDs. You need to understand what it is like being in the trenches*." While another (a clinician by background) indicated "*It isn't as important as personal skills...I could always refer them to literature or an MD."*

In contrast, those facilitation relationships or encounters perceived as not very successful were more likely characterized by interviewees and interviewers with one or more of the overlapping factors on the following list.

1. The individual who might have played an active facilitator role was more focused, per research team requirements, on project study management activities such as data collection and other evaluation duties.

2. The person who was in contact with the internal change agent/s was not introduced as, nor supported to work as a "*problem solver*." One said, *"My role got watered down. We didn't have answers to barriers so the participants got worn down, discouraged." *Another was felt to have had an inappropriate "*level of problem solving skills."*

3. Responsibility for success of the project was delegated to the sites in the form of internal change agent/s, cited as "*local champions" *or *"site leaders*," but without the support of structured QUERI facilitation, internal support, or appropriate selection.

4. Particularly in the early phases of implementation in the VHA, facilitation was not consistently addressed nor supported in an explicit, structured manner at a project team level.

## Study limitations

This evaluation was both small scale and reliant on self-report data, thus potentially limiting the generalizability of findings. Additionally, its purposively sampled participants represented a specific perspective and are likely EBP enthusiasts, particularly in terms of facilitation. However, data and findings would suggest that interviewees were able to reflect and report their experiences in a considered and balanced way; and the authors have attempted to provide sufficient information, in terms of methods, findings and context, to enable readers to make a judgment as to the rigor of the approach and transferability of findings to settings with which they are familiar. The dual role of ML as an interviewee and note taker was a potential source of bias, but a number of steps were taken to minimize this possible effect. For example, ML was interviewed first and took a mainly passive role in interviews. Furthermore, an iterative affirmation process was employed throughout the evaluation [see Table 3], and a third person acted as a critical reader, challenging or affirming data collection as well as analysis processes and outcomes.

## Discussion

Several authors have noted that the concept of facilitation has yet to be thoroughly defined and, thus, is challenging to integrate effectively into implementation research [3,9,16]. The QUERI reflective evaluation reported here, despite its limited scope, provides sufficiently suggestive findings from its multiple implementation experiences to highlight key aspects of the concept of facilitation – both in relation to the existing literature and the design of future implementation projects within and beyond the VHA.

### Purpose, nature and role of facilitation

Interviewees from QUERI discussed the purpose of their facilitation efforts – actual and as desired in the future – in terms of interactive problem solving to meet specific implementation goals. This finding is consistent with Roger's definition of a change agent [5], as "an individual who influences clients' innovation decisions in a direction deemed desirable by a change agency." Therefore, it is different from facilitating a change that involves open-ended implementation goals and objectives.

The QUERI description of facilitation as a form of interactive problem solving also is consistent with a recently published concept analysis by Thompson, et al. [18] that identified the facilitator role as active, dynamic and task-oriented. Furthermore, interviewees' emphasis upon the criticality of the role's support element reinforces findings of both Harvey, et al.'s concept analysis that concluded "the facilitator role is about supporting people to change their practice [p.585,9]," as well as recent empirical work by Cheater, et al[17] and Wallin, et al. [16].

In terms of the PARIHS definition and purpose of facilitation, there is an obvious overlap with this study's findings relative to the concept of a "helping and enabling" role [7,8]. The heuristic definition that emerged from QUERI experiences further emphasizes what PARIHS would call the "task orientated approach" but provides more explicit operational detail about the "focused process of providing help and support to achieve a specific task [p.177, [8]]." Unlike the 2002 PARIHS description, QUERI facilitators at times did use "telling or persuading," given the goal of the external change agency.

In general, QUERI interviewees felt that facilitation could be a distinct entity, but questions still remain as to exactly how this problem solving and supportive function sets facilitators apart from other change agent roles, such as educational outreach/academic detail workers, opinion leaders, or champions. To some extent the findings from this evaluation support Harvey, et al. [9] in that QUERI interviewees described their facilitation role as broader than the educational outreach model; i.e., because as facilitators they engaged in more, and differently targeted interventions. For example, interviewees described their role in diagnosing and modifying contextual factors, and in being a boundary spanner/intermediary between organizations and other stakeholders. Arguably, educational outreach workers tend not to focus their efforts on context or on providing a mediating function.

Additionally, there may be some overlap between facilitation and project management. While participants in this study viewed their facilitation role as distinct from a project management role, in reality there may at times be blurring of role boundaries and tasks undertaken. According to the findings of this evaluation and those of Harvey, et al. [9], the distinction between a facilitation intervention and project management role seems to be one of intention and scope. A facilitation intervention, for example, is concerned with enabling the implementation of evidence into practice using a wide repertoire of skills and a flexible approach to working with individuals and teams in an enabling way. On the other hand, project management is not necessarily about enabling the process of evidence implementation and is potentially more restrictive in its scope, remit and enactment. More research is obviously needed to determine the specific differences between facilitation and project management roles.

In Thompson, et al.'s concept analysis [18], various roles of "intermediaries and influentials" in the transfer of knowledge were reviewed, revealing persistent confusion regarding both standard definitions of individual roles and cross-role similarities/differences. In terms of the roles reviewed – i.e., opinion leaders, facilitators, champions, linking agents and change agents – Thompson, et al. concluded that these specific "concepts may indeed be similar phenomena with different labels [p. 691, [18]]."

A facilitator in the QUERI study clearly can be defined as a change agent [5], as well as a linking agent between the internal change agent and the broader environment. However, as Thompson, et al. also found, "there are...many differences that suggest that these [reviewed] concepts are conceptually unique [p. 691, [18]]." For example, external QUERI facilitators did not conform to various descriptions of either an opinion leader (i.e., an internal individual who is informally well-connected and has a wide peer and social network) or a champion (i.e., an individual who emerges unsolicited and has visionary qualities).

### Personal attributes

A number of individual factors were highlighted by interviewees believed critical to the success of a facilitator's role. This included flexibility, relevant experience (as facilitators or as clinical process experts within the VHA), knowledge (e.g., regarding the evidence), and the ability to build relationships through good communication. The facilitators in this study were required to be flexible by adopting different styles (e.g., directive and non-directive) depending on the projects, specific sites, related progress, and individuals involved. These findings support others' work. For example, Cheater, et al. [17] found that flexibility and the ability to draw on a repertoire of skills supported the functioning of the facilitation role in an exploratory trial. Additionally, Wallin, et al. [16] describe the positive benefits of guideline implementation facilitators having knowledge about managing change, as well as having insight into the clinical topic, which they call being 'content aware.' Furthermore, Greenhalgh, et al.'s observations regarding external change agents [6] suggest that facilitators need to have credibility and be appropriately "trained and supported to develop... interpersonal relationships with potential users [p.26]." Thompson, et al.'s review generally echoes these observations [18].

In summary, and as Harvey, et al. [9] surmise, collective findings to date, including this evaluation, indicate that to be effective, facilitators need to be flexible and possess a range of skills to be used according to the needs of those with whom they are working and the related context.

### Facilitators and barriers

A number of facilitating and hindering factors were identified in this evaluation, including the need for protected time, leadership support, and recognition of the importance of the facilitation role. The issue of protected time is one seen frequently in the evidence into practice implementation literature [10,19]. Clearly the resolution of such issues is not straightforward, and it would be naïve to suggest that just providing protected time for facilitators would enhance the success of projects. In the case of this evaluation, perhaps protected time was an indicator of the degree of organizational investment or support. For those project teams that did not understand or value the facilitator role, this resource was not forthcoming and, thus, was unavailable to help optimize change.

## Conclusion

Facilitation is a word and concept that is used frequently, yet poorly understood. There are few explicit descriptions or rigorous evaluations of the concept within the field of implementation. The findings of this small-scale, reflective evaluation suggest that facilitation could be considered a well-planned, proactive, supportive and mediating change agent role, and thus a discrete intervention that can enable site activation and enhance implementation of new, evidence-based practices. However, there are many questions that remain outstanding regarding the nature and effectiveness of facilitation. Table 8 lists one set of research questions that emerged from this evaluative study. In the future, facilitation needs to be explicitly studied both as an internal and external role intervention to better define its specific contribution to the success of implementation in different types of projects, different types of context, and with evidence and innovations of different levels of complexity. Its potential as one of the key ingredients in successful implementation, as the PARIHS model suggests, might then be realized.

**Table 8:** Future study questions regarding the concept of facilitation

1. To what extent does facilitation mediate the value of other implementation interventions?
2. To what extent does a facilitator role, compared to other implementation interventions, enhance the success of implementation? What is its cost-effectiveness?
3. Can good facilitation, in the form of an external role, help to overcome poor internal leadership?
4. What activities are crucial to the usefulness of external facilitation across different types of sites and projects?
5. Is there a toolkit that would enhance the enactment and implementation of a facilitator role?
6. How can facilitation activities be measured and related to implementation outcomes? [11]
7. Does "more facilitation" result in better implementation outcomes? Under what circumstances? Is there a "dose" effect, and, what constitutes "sufficient" facilitation activities? [11]
8. Are there stages of facilitation activities? If so, are these stages associated with and needed within the different stages of change? [20]
9. What are the similarities of and differences between a facilitator role and a project manager, consultant, and other change agent roles?
10. How does being part of an implementation study team affect the external facilitator role and project effectiveness?
11. What aspects of the external facilitator study role must be replicated by clinical leadership when they plan to use the results of a successful implementation study?

## Competing interests

The author(s) declare that they have no competing interests.

## Authors' contributions

CBS, with MWL, conceived of the study. She also participated in its design, coordination, data collection and analysis, and drafted the initial form and all revisions of the manuscript. MWL conceived of the study, with CBS, participated in its design, coordination, data collection and analysis, and also provided initial and final refinements to the manuscript. JR-M participated in its design, analysis and conceptual interpretations, drafted components of the manuscript, and provided input into initial and final refinements of the total manuscript. All other authors participated in the study's design, provided case information and feedback on iterative forms of the analysis/evolving paper, and read and agreed to the final manuscript.

## Supplementary Material

Additional File 1 *Study Projects per QUERI Team with Implementation Interventions other than Facilitation*. Narrative in single row table format Click here for file

Additional File 2 *Reflective Questions and Interviewee Preparatory Work*. Straight narrative, plus one small table and one text box. Click here for file
