# Supplementary material for: Role of "external facilitation" in implementation of research findings: a qualitative evaluation of facilitation experiences in the Veterans Health Administration
Source: Implement Sci. 2006 Oct 18;1:23. doi: 10.1186/1748-5908-1-23 (PMC1635058; doi:10.1186/1748-5908-1-23)
Supplement: Additional File 1 — Study Projects per QUERI Team with Implementation Interventions other than Facilitation. Narrative in single row table format [file 1748-5908-1-23-S1.pdf]

STUDY PROJECTS PER QUERI TEAM:
GOALS AND RELATED IMPLEMENTATION INTERVENTIONS

SCI QUERI GOAL: *Increase vaccination rates for influenza and pneumococcal pneumonia in veterans with spinal cord injury or disorders.*	o Patient reminder letters with educational materials.<br>o Practitioner education materials.<br>o Computerized-clinical reminders.<br>o Standing orders for administration of flu vaccine by nursing staff.<br>o Feedback to sites once a year on staff and patient influenza vaccination rates.

HIV QUERI GOAL: *Implement and evaluate real-time computerized clinical reminders and a collaborative intensive quality improvement program.*	o Real-time computerized clinical reminders.<br>o A collaborative group-based quality improvement program, based on the Institute for Healthcare Improvement Breakthrough Series with modifications for this project.<br>o Facility-level feedback regarding clinical performance.

IHD QUERI: GOAL: *Reduce low density lipoprotein levels in patients with IHD.*	o Optional choices for the sites:<br>• Paper point-of-care reminders,<br>• Lipid clinic,<br>• Audit/feedback to clinician,<br>• Patient-education component,<br>• Electronic clinical reminder, and<br>• Templated standing orders.

MENTAL HEALTH QUERI GOAL: *Implement an evidence-based treatment model for patients with schizophrenia.*	o Performance feedback.<br>o Pocket cards.<br>o Educational kick-off/training session for champions @ each site.<br>o Opinion leaders (also known as local champions).<br>o Clinical reminders/electronic.<br>o CQI: encouraged initiation of quality improvements.

Stetler et al, 2006, *The importance of "facilitation" to the implementation of research findings*

STUDY PROJECTS PER QUERI TEAM:
GOALS AND RELATED IMPLEMENTATION INTERVENTIONS
(CONTINUED)

SUBSTANCE ABUSE QUERI:<br>GOAL:<br>*Improve concordance with four best-practices for opioid agonist therapy.*	o 1.5 day educational site visit:<br>• On evidence-based recommendations and QI process – PDSA, and<br>• Note: Used visit to meet with leadership to learn more about baseline clinic functioning.<br>o Multiple educational and quality improvement tools:<br>• Evidence summary for recommendations,<br>• Dosing algorithm,<br>• Sample contingency management plan, and<br>• 14-item questionnaire for staff about attitudes.<br>o Monthly progress reports/audits and feedback.

SCI GUIDELINE IMPLEMENTATION<br>GOAL:<br>*Implement and evaluate the use of two new SCI clinical practice guidelines about deep vein thrombosis and bowel care.*	o Local opinion leaders (aka 'clinical champions'; site coordinators).<br>o Educational sessions:<br>• Ongoing educational in-services, and<br>• Per focus groups that also served to facilitate engagement/buy-in.<br>o Patient-mediated interventions, per mailed informational and educational brochures.<br>o Standardized documentation templates/standing orders (as prompts and reminder).<br>o Social marketing/outreach visits via a centralized clinical champion/expert.<br>o Policy change regarding retention of documentation.
