# Supplementary material for: Role of "external facilitation" in implementation of research findings: a qualitative evaluation of facilitation experiences in the Veterans Health Administration
Source: Implement Sci. 2006 Oct 18;1:23. doi: 10.1186/1748-5908-1-23 (PMC1635058; doi:10.1186/1748-5908-1-23)
Supplement: Additional File 2 — Reflective Questions and Interviewee Preparatory Work. Straight narrative, plus one small table and one text box. [file 1748-5908-1-23-S2.pdf]

<u>REFLECTIVE QUESTIONS AND INTERVIEWEE PREPARATORY WORK</u>

I. Preparation for the Interview:

1. To stimulate recall:
   - Review the interview questions (Part II below),
   - Review materials and data from their completed implementation studies, and
   - Complete 9 scaled questions about facilitation based on the PARIHS model [8].

Imagine the facilitation role you may take on in a <u>future</u> implementation project. Consider whether you agree or disagree with the following statement.

	Strongly disagree				*Strongly agree*
1. The primary purpose of this implementation role is to achieve tasks.	1	2	3	4	5
2. My clinical credibility is not important to the success of such a role.	1	2	3	4	5
3. The primary purpose of this implementation role is concerned with developing and empowering individuals and teams.	1	2	3	4	5
4. Being flexible is a key ability in this implementation role.	1	2	3	4	5
5. The project protocol would need to allow me sufficient contact time with individuals and teams.	1	2	3	4	5
6. Problem solving would be the least important activity in that role.	1	2	3	4	5
7. The formal training that I have had is not sufficient to fulfill the requirements of this implementation role.	1	2	3	4	5
8. I feel I would be empowered in this implementation role.	1	2	3	4	5
9. During such a project, I would build sustained relationships with people.	1	2	3	4	5

2. Provide background information for the interviewers:
   - Study abstract,
   - Number of study sites,
   - Nature of the interviewee's "facilitation" role at the beginning of the project, and
   - As available, study manuscripts.

II. Interview Schedule

1. Can you begin by briefly telling us about significant aspects of your Project, or if there was anything you would like to add to the summary/abstract you've given us?

2. How would you sum up your role within your *last* implementation study project? How would you "title" it?

3. Who were the other key players (roles) in this implementation project?
   a) On your study team?
   b) Within the intervention sites?

4. Now we would like to get to the detailed aspects of your role (or roles) within this implementation project. Can you tell us very specifically what it was you did in this implementation project?
   a) What were your responsibilities?
   b) Did they change over time? If so, why?
   c) Did this differ from site to site? If so, why?
   d) Did any other individual/s in your Implementation study team engage in any of these activities? (What was their study role and what did they do, exactly?)

THE NEXT SECTION DEALS WITH EXTERNAL FACILITATION (Q. 5-15) (*See definitions on page 1*[1]):

5. In terms of the activities/functions you have just described, which would you categorize as *facilitation*, as opposed to other change-related roles, such as those listed in the box on your right?

6. In terms of the behaviors/activities that you just cited as *facilitation*, which do you believe were the *most important* or *critical*, and why?
   a) Did this differ per site? If yes, how so?
   b) Who were the targets of each of these facilitation efforts?

7. In terms of these most important or critical *facilitation activities*, please think about each - in terms of the following rating scale. Can you judge any of the activities/functions in terms of the *degree of success* of your facilitative behavior?

Not at all effective 1 2 3 4 5 Very effective/successful

8. In terms of the issue of the "success" of facilitation:
   a) Overall, what was the outcome of the implementation project?
   b) Overall, what was the degree of success of your facilitation efforts?
   c) Do you have any (other) evidence of the role that facilitation might have played in the overall outcome of the implementation project?
      i) What about "barrier reduction"?

OTHER CHANGE-RELATED ROLES

Study project management: *Overseeing and organizing the overall conduct of the study, including carrying out pre-determined translation/ implementation interventions.*

Educational outreach: *Explicitly and formally teaching individuals within the study site about the best practice evidence and trying to persuade them, e.g., in face-to-face meetings, about the importance of its use. This includes helping members at the site "understand" the evidence.*

Opinion leadership: *In that individuals at the site saw YOU as an influential individual in regards to this best practice issue and related decision-making*

Championing: *In that YOU actively advocated adoption of this best practice (outside of formal educational in-services, meetings, etc.)*

Other: *General Education or Marketing*

[1] Alternative working definitions of facilitation from the PARIHS model and QUERI were provided. Interviewees were asked to use them to help "think about specific activities and role behaviors that you performed during the implementation project that would fall into the global category of 'facilitation'." These definitions were generally available to interviewees prior to the reflective exercise.

9. Please clarify the relevance of *time* to your facilitation activities. For example, did the *amount* of time you spent with various sites change over time? Was there any *pattern* involved? Was there a consistent or intermittent relationship of time & influence/need? Did you have a routine schedule in terms of *frequency* of contacts? If time spent was different *per site*, why so? Can you describe time in terms of *intensity* of the facilitation? Did *intensity* or *frequency* seem to make any difference?

10. Where, if at all, did *formative evaluation* fit within or relate to your *facilitation* role as you described it above?

11. What factors *facilitated* your role as facilitator and what *hindered* it, e.g., time, skills, location, etc.?

12. What, if anything, would have been *most helpful* to you and to the sites to improve results. Please think of this in terms of facilitation that didn't actually happen in this project.

THE NEXT SECTION DEALS WITH INTERNAL FACILITATION (Q. 13-15)

13. If applicable: **Internal Facilitation:**
    a) Who played this internal facilitator role (in terms of their routine role/project position)? Did it differ per site?
    b) Please describe that individual's multi-faceted implementation role, especially the facilitative behaviors. Did it differ per site? If so, why?

14. If applicable: **Internal Facilitation:**
    a) Can you describe how you two, as facilitators, worked together as external and internal change agents?
    b) Did you, as external facilitator, have to get engaged in the development of the internal facilitator (i.e., skilling them up to become facilitators)? ___No ___Yes (Please explain)
    c) What else, if anything did you do for them?

15. If applicable: In terms of the ***internal facilitator***, could you judge the overall degree to which this role was effective/successful? (1 2 3 4 5)
    a. On what basis did you make this judgment?
    b. What factors facilitated/hindered that person's efforts?

SUMMARY QUESTIONS:

16. In this implementation project, what was the *main* role you undertook? Did it change over the time of the project? Was it facilitation?

17. What do you believe is the essence of facilitation?
    a. In your opinion, how does facilitation relate to or differ from other change agent or implementation roles? (*Please review the SOTA paper on Lessons Learned re: Facilitation. Do you consider all the examples to represent facilitation?)*

18. In your "next" implementation project (and please describe if there is one):
    a. What would you specifically do, from a facilitation point of view, to make things easier for others or to support and encourage them to change?
    b. What would you do relative to the issue of an internal facilitator?
    c. If NOT a ROLLOUT. Do you see a facilitative function as important for rollout? ___ yes ___no. Which levels? In what way?

19. Did you have any comments to share on your responses to the 9-item PARIHS related questions?
